# Morphology and Histology of the Ductus Receptaculi and Accessory Glands in the Reproductive Tract of the Female Cricket, *Teleogryllus commodus*

**DOI:** 10.1673/031.008.3501

**Published:** 2008-05-05

**Authors:** Robert Sturm

**Affiliations:** Brunnleitenweg 4l, A-506l Elsbethen, Salzburg, Austria

**Keywords:** Insect reproduction, reproductive glands, ultrastructure, Orthoptera, Gryllidae

## Abstract

The morphology and histology of the ductus receptaculi and accessory glands in females of the black field cricket, *Teleogryllus commodus* Walker (Orthoptera: Gryllidae) are described. Both are reproductive organs situated in the 7^th^ and 8^th^ abdominal segment that join the posterior part of the genital chamber. The ductus receptaculi is a long (up to 25 mm) homogeneous tube, and the accessory glands (total length: 4 to 12 mm) are a complex system of tubes and end lobes with various numbers of ramifications. Based on their external shapes the accessory glands may be subdivided into three distinct regions, a distal region mainly producing the gland's secretion, a middle conducting region, and a basal region serving for the storage and release of the secretory substances into the genital chamber of the female. In histological respects, both organs have an outer muscle coat followed by a basal lamina, one or two cell layers, the cuticular intima, and the inner lumen. The ductus receptaculi is subdivided into three histologically different regions. The region located adjacent to the receptaculum and the region neighbouring the terminal papilla consist of a single, epithelial cell layer that is not secretory. The epithelium of the middle region contains two cell layers, glandular cells and cuticula-forming cells, which are responsible for the production of the cuticular intima. The secretion of the gland cells is released into an extracellular cavity, through which it reaches the lumen via a complex network of canals running through the intima. The histology of the accessory glands is rather homogeneous among the different regions, as one layer of epithelial cells produces both the secretion and the cuticular intima. Histological variations in the distal, middle, and basal gland sections mainly concern the height of the epithelium, the thickness of the basal lamina and the cuticular intima as well as the variable presence of the outer muscle coat. In contrast to the ductus receptaculi, secretory substances produced by the accessory gland cells accumulate in the lumen by a diffusive permeation of the intima.

## Introduction

The reproductive system of female insects commonly includes a pair of ovaries, from which the lateral oviducts emanate. In the distal segments of the abdomen these tubular structures join to form the common oviduct penetrating the posterior wall of the genital chamber. In addition to these basic components a spermatheca serves for the storage of spermatozoa transferred from the male during copulation, and paired accessory glands, which in some cases are also termed ‘collateral glands’, support the egg-laying process by producing various kinds of oily or sticky secretions (Wigglesworth 1963; Gillott 1988; Chapman 1998). Among the various insect orders, the external shape and morphology of organs in the female genital tract are variable. While the ovaries are either panoistic or meroistic, the spermatheca, being of ectodermal origin, may consist of a highly variable number of receptacular complexes, which themselves include the receptaculum seminis for the storage of the spermatozoa and the ductus receptaculi for the directed transport of the germ cells. In numerous flies, for instance, three receptacular complexes with their ducts opening either into the common oviduct or into the vagina occur, whereas in grasshoppers, crickets, and other representatives of the Orthoptera only a single receptacular complex is present, whose duct directly opens into the genital chamber (Essler et al. 1992; Chapman 1998; Sturm 2002). The accessory glands and the spermatheca are ectodermal in origin (Gillott 1988; Kaulenas 1992). While the external shape of these structures varies among different insect orders, and also within a limited group of insect species, the internal morphology strongly depends upon the function of the organs (Brunet 1952; Gillott 1988; Kaulenas 1992).

Within the order of the Orthoptera, anatomical and morphological investigations of the female reproductive system mainly concentrated upon the family Gryllidae. While early studies of the female genital tract in gryllids date back to the first half of the 20th century (Spann 1934; Snodgrass 1935, 1937), detailed scientific works on single reproductive organs including light- and electron-microscopic techniques were mainly conducted during the past three decades (King and Akai 1984; Adiyodi and Adiyodi 1988). From the results of the studies carried out more recently it could be concluded that the tubular structures of the reproductive system, such as the ductus recptaculi and accessory glands, have a basic construction scheme. Further, these structures are characterized by a subdivision into several functionally different regions, which complicated understanding and interpretation of them.

In this study, the morphology and fine structure of the ductus receptaculi and accessory glands in females of *Teleogryllus commodus* Walker (Orthoptera: Gryllidae) are compared. Besides the working out of morphological similarities as well as respective discrepancies, the results should help to extend our functional knowledge of these important structures.

## Materials and Methods

Ten to 12 day-old females of the black field cricket *T. commodus* were used. The animals, which mainly occur in the Mediterranean climate zones of Australia and New Zealand (Walker and Masaki 1989; Blank et al. 1988), were reared in a climate chamber at the Institute of Zoology, University of Salzburg, using the following setup protocol: a constant mean air temperature of 25 °C, a relative humidity of 60 ± 10 %, and a photoperiod of 12 h. While larval instars were kept in plastic boxes about 50 × 30 × 30 cm in size, adults were separated by gender and kept separately in 5 L glass vessels (Musiol et al. 1990). Animals were fed with fresh salad, standard diet for rodents (Altromin 1222, www.altromin.de), and water. Structures of interest were isolated by anesthetizing females in a CO_2_ stream, transferring them into insect Ringer's solution (pH 7.2), decapitating, and opening the ventral side of the abdomen (Prillhofer 1995). For light microscopy, organs and Ringer's solution were transferred on a glass slide and carefully covered with a thin cover slip. To obtain better contrast of single cell compartments, preparations were stained using Karmin Acetic acid (Sturm and Pohlhammer 2000). For transmission electron microscopy, the ductus receptaculi and accessory glands were fixed in a solution of 2 % paraformaldelhyde and 2.5 % glutaraldehyde (Karnovsky 1965). Both fixatives were buffered in sodium-cacodylate (0.15 M) at pH 7.4. Calibration of fixative and buffer to about 325 mOsm was conducted with sucrose. After immersion for 3 h, the fixed organs were washed in the sodium-cacodylate buffer for several times and post-fixed in 1 % O_s_O_4_ for 2 h. The specimens were washed in buffer and dehydrated in an graded series of ethanol. They were then preinfiltrated in graded mixtures of propylene-oxide and the expoxy resin embedding medium. Polymerization took place for 24 h at 40 °C followed by 24 °C at 60 °C. Semithin sections (1 to 2 µm) produced with a Reichert OM-U2 microtome were mounted on a glass slide and stained with methylene blue for light microscopy. For the subsequent electron microscopic work, ultrathin sections (200 nm) produced with the same apparatus were stained in uranyl acetate and lead citrate (Reynolds 1963). Microscopy was carried out with a Philips EM-300 electron microscope at an accelerating voltage of 80 kV.

## Results

**Figure 1.**
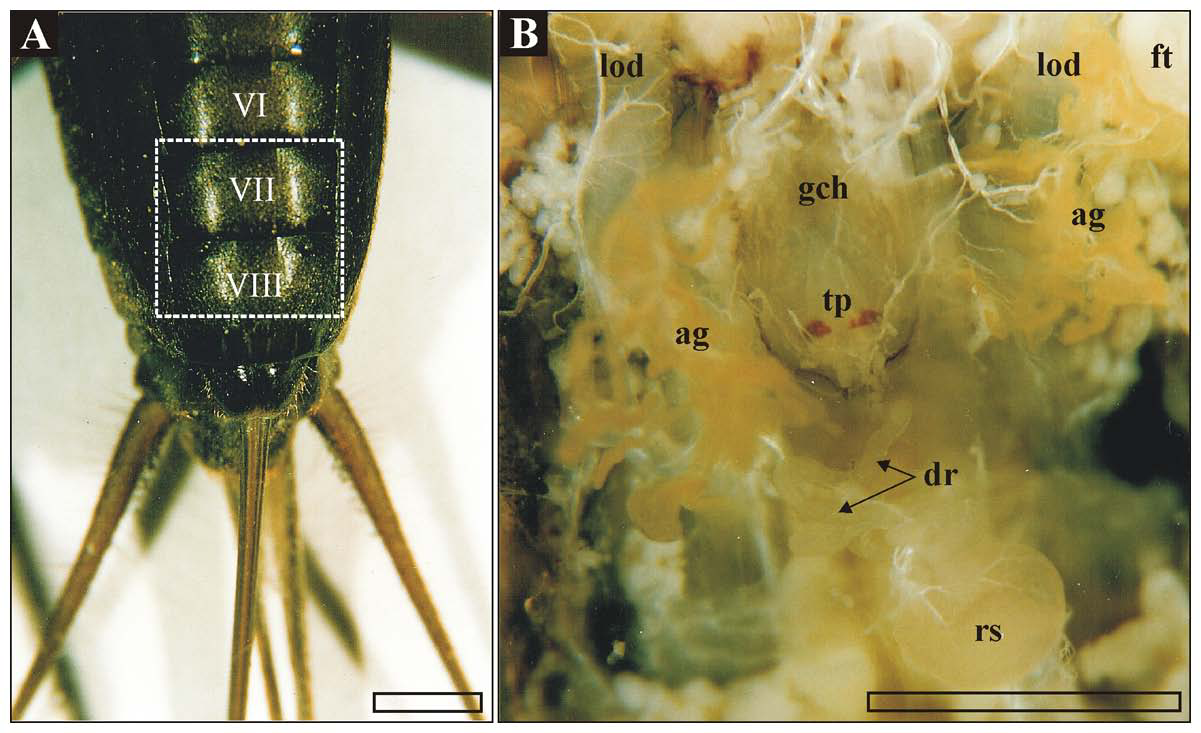
A) Terminal abdominal segments (ventral view) of female *T. commodus*. Segments containing the genital chamber and associated glands of the reproductive system are marked by Roman numbers, while the dashed frame indicates the position of the organs exhibited in image B. B) In-vivo position of selected reproductive organs in *T. commodus*. Abbreviations: ag, accessory glands; bl, basal lamina; cb, cell border; cfc, cuticula-forming cell; ci, cuticular intima; cod, common oviduct; dm, desmosome; dr, ductus receptaculi; dr_2_, ductus receptaculi, Region III; dr_3_, ductus receptaculi, Region III; ecs, extracellular space; ed, efferent ductule; ep, epithelium; ft, fatty tissue; gch, genital chamber; gc, glandular cell; int, cellular interdigitations; lod, lateral oviduct; l, lumen; mc, muscle coat; mit, mitochondrium; mt, muscle tissue; mv, microvilli; n, nucleus; nl, nucleolus; op, ovipositor; rs, receptaculum seminis; ser, smooth endoplasmatic reticulum; tp, terminal papilla; ves, vesicle. Bars: l mm.

**Figure 2.**
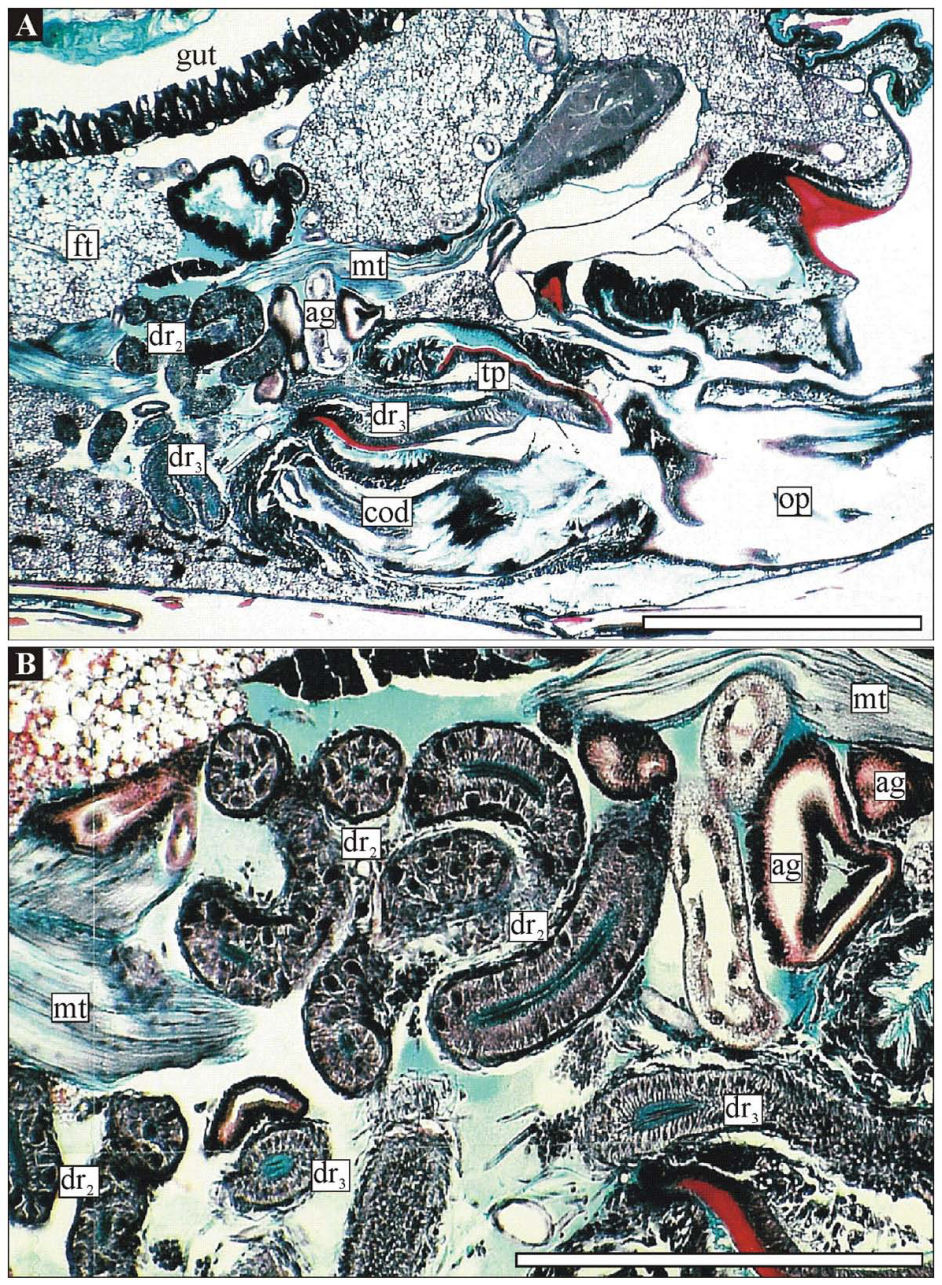
A) Median section through the abdominal genital chamber and associated structures in female *T. commodus*. B) Detailed view on the associated organs (ductus receptaculi and accessory glands). Abbreviations: see Figure I legend. Bars: I mm in A and 0.2 mm in B.

The reproductive structures are in the ventral part of the 7^th^ and 8^th^ abdominal segment of female *T. commodus*. The ductus receptaculi, representing the connective tube between the receptaculum seminis and the genital chamber, is situated directly posterior to the genital chamber and above the common oviduct (Figure 1B, Figure 2). The structure is characterized by a strongly convoluted appearance, which makes its size hard to estimate. The terminal part of the ductus forms a papilla penetrating the posterior wall of the genital chamber and is positioned directly above the seminal gutter. The paired accessory glands are marked by more lateral positions with-in the distal abdominal segments. The glands are remarkably convoluted such that they fill only a small volume of the genital tract. The organs join the genital chamber about 0.2 mm lateral to the terminal papilla (Figure 1B).

### Ductus receptaculi

**Figure 3.**
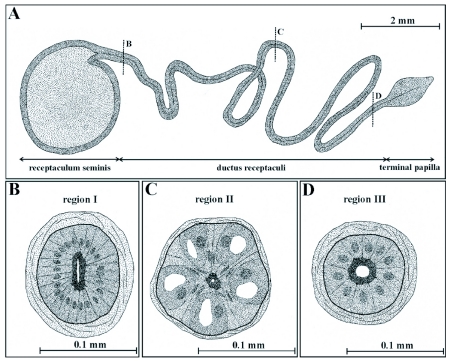
Shape and general morphology of the receptaculum seminis and ductus receptaculi in female *T. commodus*. The duct of the receptacular complex (A) may be subdivided into three regions, i. e. a region proximal to the receptaculum (Region I, B), a middle glandular region (Region II, C), and a terminal region (Region III, D). As already shown by the simplified illustrations, Region I and III includes an epithelium with only one cell type, while in Region II the epithelium is composed of two cell types fulfilling specific functions.

In gross morphology the ductus receptaculi represents a homogeneously formed tube with a constant diameter of about 0.1 mm and a length ranging from 20 to 25 mm. While the proximal part of this tubular structure opens into the receptaculum seminis, the distal part is extended to form the terminal papilla (Figure 3). The ductus receptaculi is subdivided into three functionally different regions (Region I to III) that follow a basic Bauplan consisting of an outer muscle coat, a basal lamina, one or two layers of epithelial cells, a cuticular intima, and an inner lumen. As the spermatozoa are transported through the tube a glandular secretion is released.

In Region I of the ductus, the muscle sheet reaches a thickness of about 20 µm, while the basal lamina is 0.5 to 1 µm thick. The epithelium is composed of a single layer of uniformly shaped cells, reaching a baso-apical dimension of 20 to 25 µm (Figure 2B, Figure 3B, Figure 5A). The cuticular intima varies in thickness between 7 and 8 µm and consists of three layers, a thin osmiophilic outer layer, a rather homogeneous middle layer, and a thick inner layer including chitinuous components with variable density when visualized by the electron microscope. The intima forms zipper-like extensions that allow the lumen to be narrowed and to act as an insurmountable barricade for the spermatozoa. According to Essler et al. (1992) this region of the ductus receptaculi should prevent an uncontrolled flow of spermatozoa towards the terminal papilla. While in virgins the diameter of the lumen can have a minimum diameter of about 0.5 µm, in mated females the lumen is significantly extended and has a diameter of 30 µm, allowing the passage of the spermatozoa. Regarding the epithelial cell structure of Region I, the nucleus is commonly positioned in the basal part of the cell, while the apical cell membrane is marked by the formation of a dense layer of microvilli. Adjoining cells have interdigitations that probably increase the mechanical stability of the epithelium.

Region II extends over two thirds of the ductus receptaculi and is the main section of the organ. Muscle layer and basal lamina of this region have a reduced thickness (8 µm and 0.3 µm, respectively). In contrast to Region I, the epithelium has a baso-apical dimension of about 30 µm is composed of two cell types, gland cells and cuticula-forming cells, that are arranged in two rows and are connected with the basal lamina (Figure 3C, Figure 5B-D). The cuticular intima reaches a thickness of about 6 µm and consists of an osmiophilic outer layer and an inhomogeneous inner layer. The gland cells are completely enclosed by the sheet-like processes of the cuticula-forming cells, resulting in the separation of the secretory cells from the cuticular intima and from nearest cells of the same kind. In ultrastructural respects, the cuticula-forming cells contain elongated ovoid or sickle-shaped nuclei that are situated in the apical part of the cell. The cell matrix is commonly characterized by low amounts of endoplasmatic reticulum and a very reduced number of mitochondria. The main component of the gland cell is the central cavity that is a deep invagination of the apical cell membrane (Figure 5C, D). This functionally important extracellular space is surrounded by a dense sheet of microvilli and numerous osmiophilic vesicles, indicating high secretory activity of these cells. After the release of the secretory substances into the central cavity, transport of the secretions towards the lumen of the ductus receptaculi occurs through an extracellular canal system starting inside the cavity with the efferent ductule (Figure 5D). The duct itself consists of two cuticular layers and runs from the apical top of the gland cells towards the cuticular intima forming a complex system of meanders. After entering the intima, the duct joins a network of canals that are mainly oriented parallel to the lumen of the ductus receptaculi. These canals open into the main lumen (2 to 3.5 µm in diameter) via a cleft-like orifice. In addition to the microvilli and vesicles the cytoplasm of the gland cells also contains high numbers of mitochondria as well as densely arranged endoplasmatic reticulum. The basally situated nuclei are characterized by spherical or irregular shapes and contain a polymorphous nucleolus.

**Figure 4.**
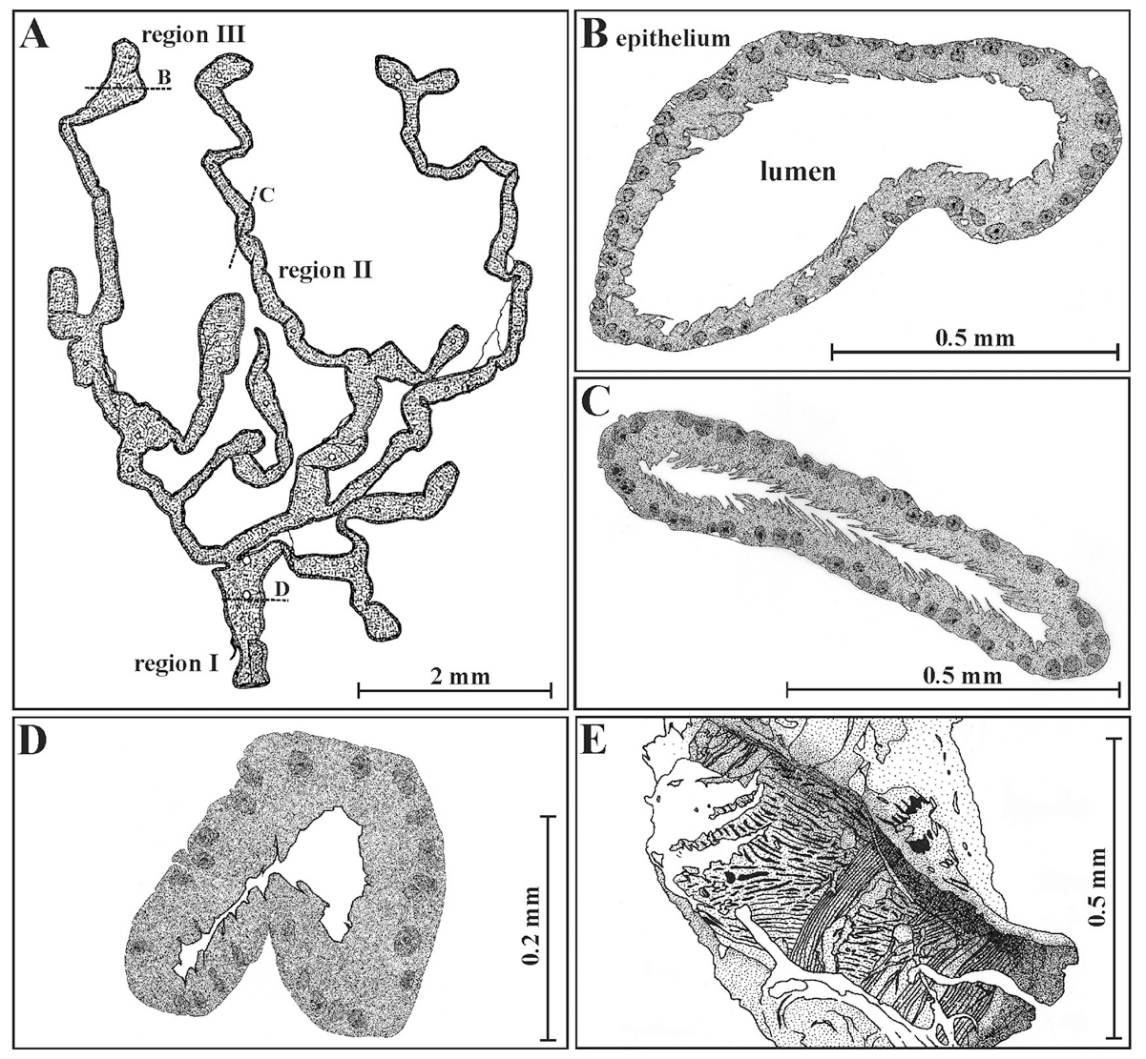
Shape and morphology of the accessory glands in female *T. commodus*. A single gland (A) is commonly characterized by a complex system of ducts and end lobes, allowing a subdivision of the organ into three regions. The basal region (Region I, D) includes the part of the gland near its orifice, the middle region (Region II, C) the ductal parts, and the apical region (Region III, B) the end lobes. Adjacent to the orifice the gland's epithelium is additionally surrounded by a muscle coat consisting of longitudinal and circular muscle fibres (E).

**Figure 5.**
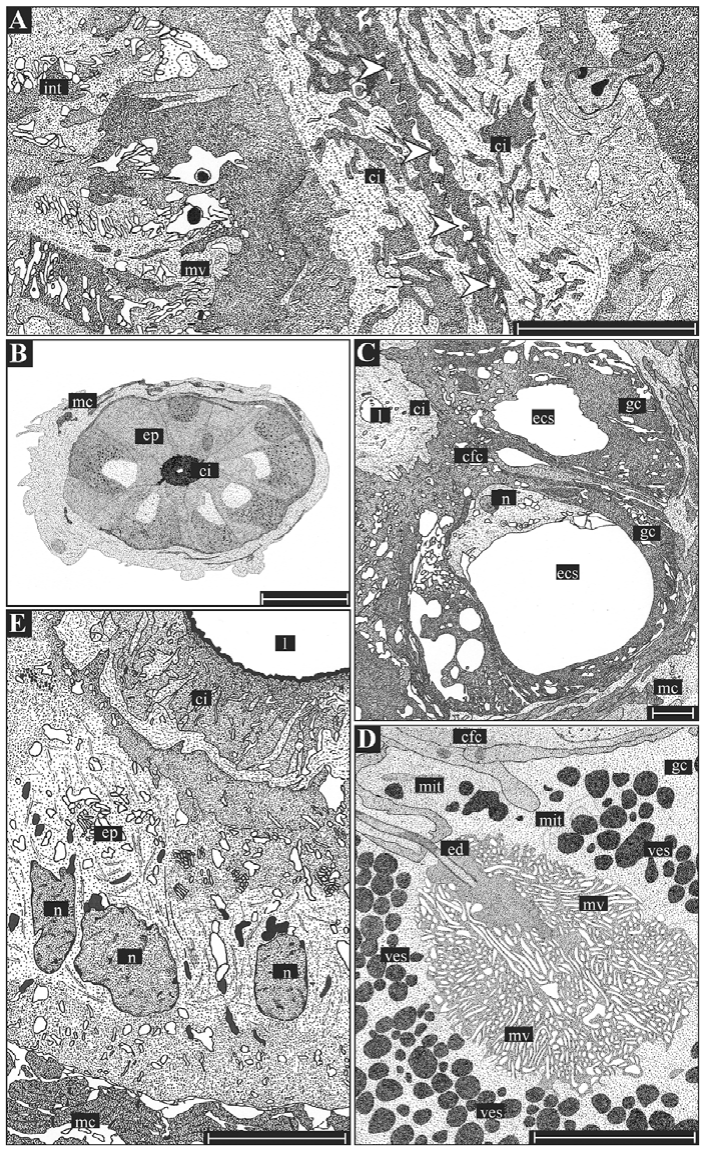
Detailed illustrations of the histology and ultrastructure of the ductus receptaculi. Region I (A) is chiefly characterized by a zipper-like longitudinal section of the lumen (arrowheads) preventing an uncontrolled outflow of spermatozoa from the receptaculum. Region II (B-D) consists of glandular cells (gc) producing the ductal secretions and cuticula-forming cells (cfc) being responsible for the formation of the cuticular intima (ci). The gland cells contain an extracellular space (ecs), into which the secretions are re leased and transported towards the intima through an efferent ductule (ed). The cavity is surrounded by a dense layer of microvilli (mv). Region III (E) consists of uniformly structured, cubic to columnar epithelial cells, an inhomogeneously structured cuticular intima, and a rather wide lumen (I). Abbreviations: see Figure I legend. Bars: 30 µm in B, 5 µm in A and C-E.

Region III extends over the terminal part of the ductus receptaculi. It is composed of a 15 µm thick muscle layer, an epithelium including only a single cell type and showing a baso-apical dimension of 10 to 15 µm, and a cuticular intima with an average thickness of 5 µm (Figure 3D, Figure 5F). In this section of the ductus receptaculi the lumen increases to a diameter of 30 µm, but, again, significantly narrows, when reaching the terminal papilla. The non-secretory epithelial cells are marked by a cubelike or slightly columnar shape and contain almost spherical or ovoid nuclei with high fractions of electron-dense heterochromatine. Mitochondria are mainly present in the apical region of the cell, while low amounts of the endoplasmatic reticulum occur. The cuticular intima has numerous groove-like structures running parallel to the lumen that are similar to the zipper-like shapes occurring in Region I.

### Accessory glands

In contrast to the perfectly tubular ductus receptaculi, the external shape of the accessory glands is characterized by complexity, with a system of highly ramified tubes and lobes. Based on their typical appearances, these organs can be subdivided into three different regions. Region I, a basal region proximal to the gland's opening into the genital chamber, Region II, a middle region consisting of flattened ductal structures, and Region III, a distal or apical region, where the ducts are extended to lobes (Figure 4A). Depending upon the grade of cellular differentiation (Sturm and Pohlhammer 2000; Sturm 2002), the dimension from the organ's base to the top of the end lobes ranges from 4 to 12 mm. Secretory activity is highest in the distal lobes, while the ducts of the middle region mainly serve for the transport of the produced secretion towards the basal part and the gland's orifice. Concerning their general morphology, the accessory glands are based on the same Bauplan as the ductus receptaculi with an outer muscle layer followed by a basal lamina, an epithelium including a single layer of secretory cells, a cuticular intima, and the glandular lumen, into which secretory substances are released.

Region I extends over the basal part of the accessory glands, and has an outer muscle sheet with an average thickness of 20 µm (Figure 4D, E). The ∼ 0.6 µm thick basal lamina consists of several thin layers. The epithelial cells reach a maximum baso-apical dimension of 40 µm. The cuticular intima has an average thickness of 6 µm and is composed of three well distinguishable layers, including an osmiophilic outer layer (0.3 µm), a darker middle layer (1.5 to 2 µm), and a brighter inner layer (4 to 5 µm) with partly randomly oriented chitinous elements. Processes formed by the intima are characterized by spine- or hair-like shapes, reaching a length of 10 µm, and are commonly oriented towards the gland's orifice (Figure 4D). The lumen has a diameter of about 50 µm but can be significantly narrowed by the contraction of the circular muscle fibres, so that a possible closing mechanism at the gland's base has been hypothesized (Sturm and Pohlhammer 2000). In morphological respects, the epithelium consists of a uniformly structured cell type being responsible for the formation of both the secretory substances and the cuticular intima. The basally situated nucleus with its frequently occurring ovoid shape reaches a maximum diameter of 15 µm and contains, besides a well-defined nucleolus, various amounts of electron-dense heterochromatin. Endoplasmatic reticulum may be detected only in low amounts, while mitochondria are present in higher number and mainly accumulate in the more apical cell region. The apical cell membrane forms a partly dense layer of microvilli. Connections between two adjacent cells are apparent either by gap junctions mainly occurring in the basal cell parts or by punctate desmosomes.

Region II of the accessory glands includes the ducts of the accessory glands that do not contain any outer muscle sheet, so that its basic architecture is reduced to a 0.4 µm thick basal lamina, an epithelium with a maximum baso-apical dimension of 45 µm, a 6 to 7 µm thick cuticular intima, and an inner lumen with a diameter ranging from 40 to 80 µm. The cuticular intima has the typical Bauplan composed of three well distinguishable layers, exhibiting the same characteristics and very similar dimensions as those mentioned for Region I (Figure 4C). In contrast to Region I, processes formed by the intima may reach a maximum length of 15 µm. Cells of the one-layer epithelium are very uniformly structured, containing a basally situated nucleus (diameter: 8–15 µm) with central nucleolus and variable contents of heterochromatin. Amounts of endoplasmatic reticulum and mitochondria are increased compared to Region I. Smooth endoplasmatic reticulum is frequently observed in high concentrations in the apical cell region, where a dense sheet of microvilli is also present. Cell-cell connections occur as shown by gap junctions and punctate desmosomes.

**Figure 6.**
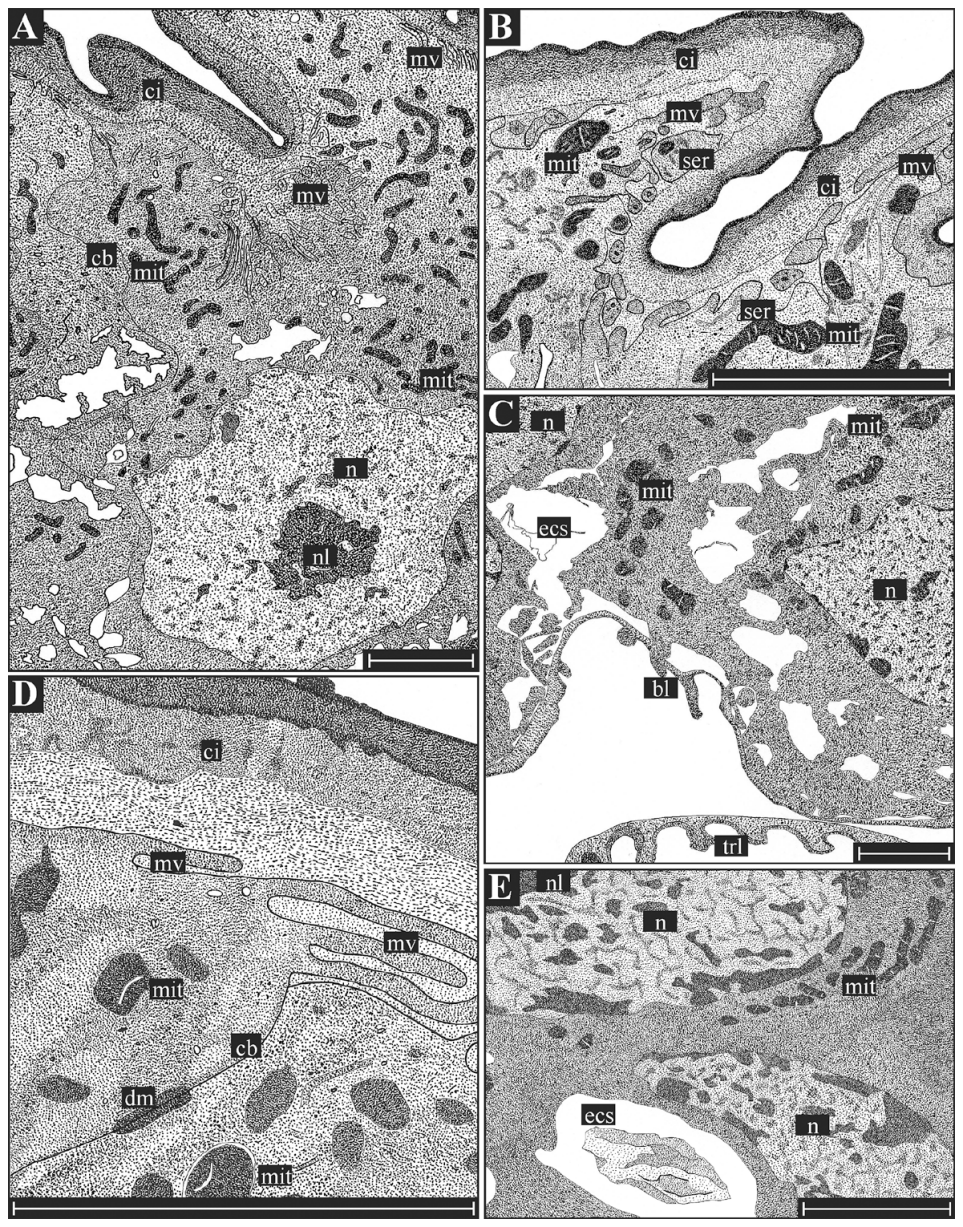
Detailed illustrations of the histology and ultrastructure of the accessory glands. As ultrastructural differences between the three externally determined regions only concern the thickness of the basal lamina and the cuticular intima, main attention is focused on the secretory-active cells of Region III. Each cell (A) may be subdivided into a basal part (C, E) containing the nucleus as well as an apical part (B) with all compartments necessary for the production of the lipophilic secretion. The cuticular intima consists of three layers, i. e. an osmiophilic outer layer, a dense middle layer, and a loosely structured inner layer (D). Abbreviations: see Figure 1 legend. Bars: 10 µm.

Region III consists of the gland's end lobes, where the major amount of secretion is produced, and is based on a similar ‘reduced’ architecture as Region II without an outer muscle sheet. The basal lamina reaches a dimension of up to 0.7 µm and is followed by a one-layer epithelium with a maximum baso-apical extension of 60 µm. The three-layer cuticular intima demarcating the epithelial cells from the glandular lumen varies in its thickness between 6 and 8 µm, whereby processes formed by this chitinous structure may reach a length of 20 µm. The lumen is significantly extended compared to Region I and II, thereby reaching a diameter of up to 300 µm (Figure 4B). Basic epithelial cell morphology of Region III does not remarkably deviate from that documented for Region I and II except that cellular compartments participating in the production of the gland's secretion are increased in number. The mitochondria, are massively accumulated in the apical region, and smooth endoplasmatic reticulum, which is chiefly responsible for the production of the glandular secretion, is present in enhanced amounts directly beneath the microvilli sheet. In some cases, single microvilli are also intruded by this essential cellular compartment (Figure 6). The basal part of an epithelial cell contains the nucleus (diameter: 10 to 15 µm), with a large nucleolus, and lower amounts of mitochondria. Sometimes the basal cell membrane, as well as the basal lamina, are invaginated into the cytoplasm, causing a significant enlargement of the basal cell surface (Figure 6C). This phenomenon is interpreted as the mark of a highly active cell taking up nutrients from the hemolymph (Sturm and Pohlhammer 2000). The lipophilic secretion produced in each cell is evenly distributed in the apical cytoplasma and penetrates the cuticular intima by simple diffusion and not by passing a specific canal system as is the case in Region II of the ductus receptaculi. At those locations, where the intima is remarkably thinned out, for example in the basal parts of long hair-like processes, a preferential evacuation of the cell's secretion into the lumen takes place.

## Discussion

In the study presented here, the morphology of the ductus receptaculi and the accessory glands in female *T. commodus*, which both represent essential structures of the reproductive system, were examined in detail. Regarding the external shape, the ductus that forms a nearly perfect tube differs from the accessory glands that consists of a complex systems of tubes, ramifications, and end lobes. Despite these differences, the secretory activity of these organs is mainly limited to certain regions, i.e. the middle Region (Region II) in the ductus and the apical region (Region III) in the accessory glands. As discussed by Essler et al. (1992), a serial system of glandular units is present in the ductus receptaculi that produce secretions that feed and prolong the life of the spermatozoa. The specific arrangement of the gland cells results in a significant extension of the glandular region of the duct and, as a consequence, of the whole structure of the duct, so that the organ's length clearly surpasses the body length of the cricket. Similar morphological results were also reported for the ductus receptaculi of *Gryllus assimilis* (Spann 1934) and *Gryllus campestris* (Dallai and Melis 1966). In the accessory glands, glandular units, being mainly concentrated in the end lobes and apical zones of the conducting region, are subject to a somewhat parallel arrangement, whereby the secretion is transported through the ducts and accumulated in the basal region of the organs. This system causes a significant shortening of the accessory structures with respect to the ductus receptaculi. Nevertheless, the organs positioned laterally relative to the genital chamber may reach a length greater than 10 mm (Sturm and Pohlhammer 2000) and thus have to be rolled up to fit into the given space of the abdominal segments. The enhanced size of the accessory glands can also be observed in females of *Gryllus bimaculatus* and *G. assimilis* (Sturm 2002), while in *Acheta domesticus* L. these structures rarely exceed a length of 3–4 mm (Sturm 2003).

Both organs have a basic construction Bauplan that has a rather wide distribution within the insects (Gillott 1988; Chapman 1998). They have an outer muscle coat consisting of several layers of muscle fibers followed by a basal lamina, one or two layers of epithelial cells, and a cuticular intima, demarcating the epithelium from the organ's lumen. In the accessory glands of *T. commodus* and other crickets, the epithelium is consistently composed of a single cell layer. According to Gillott (1988), cells within a single-layered epithelium of a glandular structure usually produce both the secretory substances and the cuticular intima. Outside the group of the orthopterans, the epithelial organization of female accessory glands is subject to a wide variation, whereby the two-layered epithelium with glandular and cuticula-forming cells is very frequently observed, for example, in *Hyalophora cecropia* (Berry 1968) and in *Periplaneta americana* (Brunet 1952; Mercer and Brunet 1959). The ductus receptaculi of *T. commodus* represents a mixture of both epithelial construction principles in so far as the proximal and terminal region both have a one-layered epithelium, whereas the middle region has a two-layered epithelium. This structural organization also occurs among other orthopterans (Essler et al. 1992), while for other insect groups the morphological data are rather scarce. Regarding the ductus receptaculi of *T. commodus*, the muscle sheet encloses the whole organ and is increased at the opening of the receptaculum seminis and at the terminal papilla. In contrast, the accessory glands have a reduced muscle coat exclusively surrounding the basal section of the organ. These differences may be due to the different functions of the muscle coat in the observed structures; while in the ductus spermatozoa are actively transported towards the receptaculum or papilla due to peristaltic contractions of the muscle fibres (Essler et al. 1992; Sturm 2005), in the accessory glands muscle fibres mainly serve for the closure of the orifice (Sturm and Pohlhammer 2000).

Regarding the cellular organization and ultrastructure of the ductus receptaculi and the accessory glands, each of the three regions of the ductus is characterized by one or two highly specific cell types, whereas the three regions of the accessory glands only include one, ubiquitous, type of cell. In the ductus, the middle region produces secretions for the transport and nutrition of the spermatozoa, while epithelia of the proximal and terminal region mainly control the flow of the reproductive cells (Essler et al. 1992). The gland cells of the ductus differ in two main respects from gland cells of the accessory glands. First, the ductal cells contain a central extracellular cavity, into which the secretion is released prior to the transport into the lumen, while accessory gland cells directly release their secretory products into the lumen. Second, ductal gland cells are demarcated from the lumen by a rather thick cuticular intima containing a complex network of canals for the evacuation of secretory products. In accessory glands, on the other hand, this cuticular intima is comparably thin and does not exhibit any canal structures (Sturm and Pohlhammer 2000). These differences in cellular structure are most likely due to the differences in solubility of the produced secretion. According to the early studies of Eidmann (1922) and Treherne (1957) as well as more recent investigations of Koeniger et al. (1996), chitinous layers are permeable to lipophilic fluids but represent an insurmountable barricade for hydrophilic secretions with enhanced contents of proteins. As demonstrated by Sturm and Pohlhammer (2000) as well as Sturm (2002), the accessory glands of *T. commodus* mainly produce oily substances being temporarily stored in the cytoplasm as fine droplets that penetrate through the cuticular intima by diffusion. The secretions of the ductus receptaculi are composed of proteins that are secreted from the producing cell through large numbers of vesicles which release their contents into the central cavity (Essler et al. 1992). The two principles of insect gland construction introduced in this study only represent a small selection of glandular architecture in insects as underlined by earlier studies of accessory glands (Brunet 1952; Berry 1968; Fuseini and Kumar 1972; Lococo and Huebner 1980) and wax glands (Waku 1978). This investigation mainly confirms the relationship between the type of secretion and gland cell structure.
